# Chronic Obstructive Pulmonary Disease and Asthma Among Workers and Residents of Navanakorn Industrial Zone, Thailand

**DOI:** 10.3390/medsci14020208

**Published:** 2026-04-23

**Authors:** Narongkorn Saiphoklang, Pitchayapa Ruchiwit, Pasitpon Vatcharavongvan, Kanyada Leelasittikul, Apiwat Pugongchai, Orapan Poachanukoon

**Affiliations:** 1 Department of Internal Medicine, Faculty of Medicine, Thammasat University, Pathum Thani 12120, Thailand; toon.pitchayapa@gmail.com; 2 Medical Diagnostics Unit, Thammasat University Hospital, Pathum Thani 12120, Thailand; lee.kanyada@gmail.com (K.L.); pu.apiwat@gmail.com (A.P.); 3 Center of Excellence for Allergy, Asthma and Pulmonary Diseases, Thammasat University Hospital, Pathum Thani 12120, Thailand; orapanpoachanukoon@yahoo.com; 4 Department of Community Medicine and Family Medicine, Faculty of Medicine, Thammasat University, Pathum Thani 12120, Thailand; pasitpon@tu.ac.th; 5 Department of Pediatrics, Faculty of Medicine, Thammasat University, Pathum Thani 12120, Thailand

**Keywords:** asthma, chronic obstructive pulmonary disease, industrial zone, obstruction, pulmonary function

## Abstract

**Background:** Industrial activities may contribute to airway diseases, particularly chronic obstructive pulmonary disease (COPD) and asthma, which are major respiratory health problems with geographically variable prevalence. The objective of this study was to assess the prevalence of COPD and asthma and to examine factors associated with impaired pulmonary function among workers and residents of the Navanakorn Industrial Zone, Thailand. **Methods:** A cross-sectional study was performed from September 2025 to January 2026 among adults aged ≥18 years who were employed in or residing within the Navanakorn Industrial Zone. Data collected included demographic characteristics, comorbidities, respiratory symptoms, chest radiographic findings, and spirometric parameters, including forced vital capacity (FVC), forced expiratory volume in one second (FEV_1_), and bronchodilator responsiveness (BDR). COPD was defined as the presence of respiratory symptoms in conjunction with at least one risk factor and a post-bronchodilator FEV_1_/FVC < 70%. Asthma was defined by the presence of respiratory symptoms with a positive BDR. **Results:** Among the 373 participants (65.4% female; mean age 55.0 ± 13.6 years), the prevalence of COPD and asthma was 4.3% and 5.4%, respectively. Abnormal chest radiographic findings were present in 8.6%, while abnormal pulmonary function was identified in 30.8%. Lung function abnormalities included airway obstruction (12.9%), restrictive patterns (9.7%), mixed defects (2.1%), and small airway disease (6.2%). A positive BDR was detected in 2.4% of participants. Multivariable logistic regression analysis demonstrated older age, male sex, a history of asthma, and the presence of chest tightness as independent predictors of abnormal lung function. **Conclusions:** COPD and asthma were prevalent among individuals working or living in the industrial zone, and abnormal pulmonary function—particularly obstructive defects—was common. Older age, male sex, a history of asthma, and respiratory symptoms were associated with a greater risk of lung function impairment, underscoring the importance of targeted surveillance and preventive strategies in industrial environments.

## 1. Introduction

Occupational lung diseases are common among industrial workers and result from prolonged exposure to harmful substances, including chemicals, dust, and airborne pollutants. The afflictions encompass chronic obstructive pulmonary disease (COPD), asthma, interstitial lung disease, silicosis, and pneumoconiosis, which can lead to progressive lung injury and impaired respiratory function [1,2,3].

The Navanakorn Industrial Zone is one of the major industrial estates in central Thailand, comprising a diverse range of industries, including electronics and electrical appliance manufacturing, automotive parts production, plastics and polymer processing, chemical and petrochemical industries, and food processing. These industrial activities are potential sources of various airborne pollutants, such as particulate matter, volatile organic compounds (VOCs), and chemical sensitizers, which may contribute to adverse respiratory health outcomes among workers and nearby residents.

Asthma prevalence may be particularly elevated among workers engaged in artificial stone manufacturing, especially those exposed to phthalic anhydride and epoxy resins [2]. In the general adult population of Thailand, asthma prevalence has been reported to range from 3% to 10% [4,5]. Globally, occupational asthma accounts for approximately 5–25% of adult-onset asthma cases [6,7,8], and occupational exposures are frequently underrecognized contributors to asthma development in adulthood [8].

COPD and asthma are highly prevalent respiratory conditions that pose major public health challenges worldwide, including Thailand. Increasing urbanization, traffic-related emissions, industrial pollution, biomass and agricultural waste burning, as well as forest fires, have contributed to deteriorating air quality, leading to an increased disease burden among individuals with COPD and asthma, particularly through elevated levels of fine particulate matter (PM2.5) [1,9]. Globally, COPD is a leading cause of mortality, predominantly affecting older adults and individuals with long-term exposure to tobacco smoke or environmental pollutants [9]. Epidemiological studies in Thailand have estimated COPD prevalence to be approximately 4–8% among adults [5,10].

Disease progression is often accelerated in untreated individuals and may be complicated by acute exacerbations requiring hospitalization, thereby contributing to increased healthcare utilization, reduced quality of life, and substantial economic burden for patients and their families [11,12]. While early detection through community-based screening may facilitate identification of individuals with undiagnosed respiratory disease and enable timely clinical evaluation and management, the role of population-based screening for COPD remains controversial. Current evidence suggests that routine screening of asymptomatic individuals has limited impact on disease outcomes, and the most effective intervention to slow lung function decline is the reduction or elimination of causative exposures, particularly smoking cessation [13,14,15,16]. Therefore, early detection strategies should be interpreted cautiously and are likely to be most beneficial when targeted toward high-risk populations, combined with interventions addressing modifiable risk factors and appropriate long-term management [13,14,16].

Given the limited epidemiological data on airway diseases in industrial zones of Thailand, this study was undertaken to estimate the prevalence of COPD and asthma and to identify factors associated with abnormal pulmonary function among workers and residents of the Navanakorn Industrial Zone.

## 2. Materials and Methods

### 2.1. Study Design and Participants

A cross-sectional study was performed from September 2025 to January 2026 among workers and residents of the Navanakorn Industrial Zone, Pathumthani Province, Thailand. The Navanakorn Industrial Zone is located in central Thailand, approximately 40 km north of Bangkok. Participants were recruited through community outreach and workplace-based invitations, including announcements at industrial facilities and nearby residential areas. Individuals who were interested in participating voluntarily presented for screening and enrollment; therefore, the study population consisted of a convenience sample rather than a randomly selected population. The sample included both industrial workers and residents living in proximity to the industrial zone, representing a mixed population with potential occupational and environmental exposures. The total population of the Navanakorn Industrial Zone, including both workers and residents, is estimated to be approximately 269,800. Based on this estimate, the proportion of individuals included in this study can be approximated to provide context regarding the representativeness of the study sample. However, proportion of the participants who were workers and residents was not precisely classified.

Eligible participants were adults aged ≥18 years. Exclusion criteria included active respiratory infection, recent myocardial infarction, uncontrolled hypertension (blood pressure >180/100 mmHg), resting heart rate >120 beats per minute, pregnancy, and inability to perform acceptable spirometry.

The study protocol was approved by the Human Research Ethics Committee of Thammasat University (Medicine) (IRB No. MTU-EC-IM-1-024/68; COA No. 106/2568; date of approval: 19 May 2025). Written informed consent was obtained from all participants prior to enrollment. The study was prospectively registered with ClinicalTrials.gov (NCT07383896) accessed on 18 January 2026.

### 2.2. Procedures and Outcomes

Data collected included demographic characteristics, underlying comorbidities, respiratory symptoms, chest radiographic findings, and pulmonary function parameters obtained via spirometry. These parameters comprised forced vital capacity (FVC), forced expiratory volume in one second (FEV_1_), peak expiratory flow (PEF), forced expiration flow rate at 25–75% of FVC (FEF_25–75_), and bronchodilator responsiveness (BDR).

Spirometry was conducted in accordance with established international guidelines from the United States and Europe [17,18,19] using a PC-based spirometer (Vyntus SPIRO, Vyaire Medical, Mettawa, IL, USA). Participants were instructed to perform a maximal inhalation followed by a forceful and rapid exhalation into the mouthpiece, sustaining the maneuver for at least 15 s. FVC, FEV_1_, FEV_1_/FVC, PEF and FEF_25–75_ were expressed in liters (L), percent predicted (%pred), percentages (%), or liters per second (L/s), as appropriate. Predicted values were established using the Global Lung Function Initiative (GLI) reference equations [20]. BDR testing was performed by administering 400 µg of salbutamol, with repeat spirometry conducted 15 min after inhalation [17,18,19].

Baseline lung function was categorized using standardized criteria. Airway obstruction was defined as an FEV_1_/FVC ratio below the lower limit of normal (LLN). A restrictive pattern was defined as an FEV_1_/FVC ≥ LLN in conjunction with an FVC < LLN [21]. A mixed ventilatory defect was defined as a reduced FEV_1_/FVC ratio with a concomitant reduction in FVC below the LLN. Small airway disease was defined as an FEF_25–75_ < 65% of the predicted value [22]. A positive BDR was defined as an increase in FEV_1_ or FVC of more than 10% of the predicted value following bronchodilator administration [21].

Airway diseases were classified as COPD or asthma. COPD was defined by the presence of respiratory symptoms (e.g., cough, sputum production, dyspnea, or wheezing), relevant exposure history—particularly smoking ≥10 pack-years or biomass fuel use—and a post-bronchodilator FEV_1_/FVC ratio <70% [16]. Asthma was defined as the presence of respiratory symptoms (e.g., wheezing, dyspnea, chest tightness, or cough) in combination with a positive BDR [23].

Chest radiographs were performed in all participants using the standard upright posteroanterior projection, and all images were independently reviewed by two board-certified radiologists.

### 2.3. Statistical Analysis

Prior research [24] reported the prevalence of abnormal pulmonary function among occupationally exposed individuals to be 22.6%. This prevalence was adopted as the expected rate for the present study. The required sample size for proportion estimation was determined with a statistical power of 80%, a two-sided type I error rate of 5%, and a margin of error of 5%, resulting in a minimum sample size of 269 participants.

Categorical variables were summarized as frequencies and percentages. Continuous variables were presented as mean values with corresponding standard deviations. Comparisons of categorical variables between participants with normal and abnormal pulmonary function were conducted using the chi-square test. Differences in continuous variables between the two groups were assessed using Student’s *t*-test. Multivariable logistic regression analysis was conducted to identify factors independently associated with abnormal pulmonary function. Variables such as age, sex, comorbidities, and respiratory symptoms were included in the model if they were statistically significant in univariate analyses (*p* < 0.05). Adjusted odds ratios (ORs) with corresponding 95% confidence intervals (CIs) were estimated. A two-tailed *p*-value < 0.05 was considered statistically significant. All analyses were performed using SPSS software, version 26.0 (IBM Corp., Armonk, NY, USA).

## 3. Results

**Figure 1:**
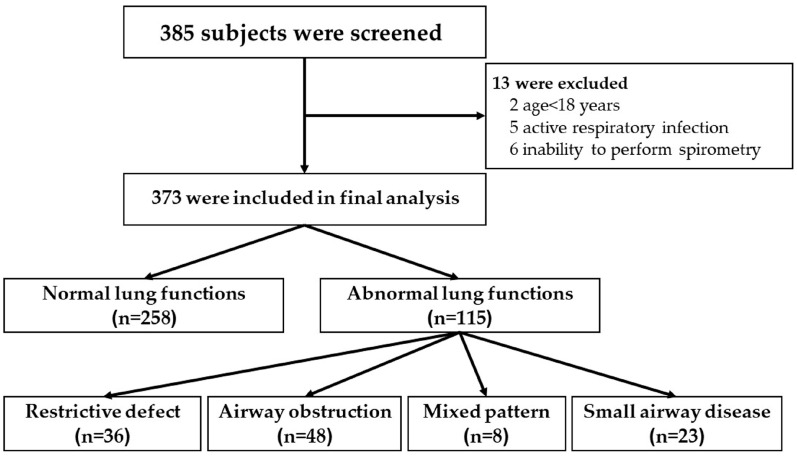
Flowchart of participant recruitment to the study.

A total of 385 participants underwent initial screening, of whom 373 participants were ultimately enrolled in the analysis, with females comprising 65.4% of the study population; 13 individuals were excluded (Figure 1). The mean age was 55.0 ± 13.6 years. Current or former smokers represented 18.2%, with a mean cumulative smoking exposure of 14.6 ± 16.8 pack-years. Employment in the textile industry and chemical industry accounted for 8.8% and 5.9%, respectively. Hypertension (29.0%) and dyslipidemia (21.2%) were the most frequently reported pre-existing comorbid conditions. The most commonly reported respiratory symptoms were cough (18.0%) and sputum production (15.5%) (Table 1).

The mean FEV_1_ was 92.5 ± 14.1% of predicted value, while the mean FEV_1_/FVC ratio was 80.41 ± 6.93% (Table 2). Overall, 30.8% of participants demonstrated abnormal pulmonary function (Table 3), comprising restrictive defect (9.7%), airway obstruction (12.9%), mixed defect (2.1%), and small airway disease (6.2%). A positive BDR was identified in 2.4% (Table 3). The prevalence of COPD and asthma was 4.3% and 5.4%, respectively (Table 2). Abnormal findings on chest radiography were detected in 8.6% of participants, including lung fibrosis (3.4%), pleural fibrosis (2.1%), lung nodules (4.8%), and mixed patterns (4.0%) (Table 3).

Compared with participants who had normal pulmonary function, those with abnormal results were significantly older and included a higher proportion of males, smokers, individuals employed in the chemical industry, and participants with hypertension, a history of asthma, cough, chest tightness, prior treatment for dyspnea, and emergency department visits for dyspnea within the preceding year (Table 1). Multivariable logistic regression analysis revealed that older age, male sex, a history of asthma, and the presence of chest tightness were independently associated with an increased likelihood of abnormal pulmonary function (Table 4).

## 4. Discussion

This cross-sectional study provides contemporary epidemiological data on abnormal pulmonary function, COPD, and asthma among workers and residents of the Navanakorn Industrial Zone, Thailand. Using standardized spirometry and internationally accepted diagnostic criteria, we found that nearly one-third (30.8%) of participants exhibited abnormal pulmonary function, with airway obstruction (12.9%) and restrictive defects (9.7%) being the most common patterns. These findings underscore the substantial burden of subclinical and overt respiratory impairment in industrial and peri-industrial communities.

Air quality in the Navanakorn Industrial Zone is likely influenced by a combination of urban traffic emissions, industrial activities, and regional transboundary pollution, similar to patterns observed across the Bangkok metropolitan region and central Thailand. Particulate matter, particularly PM_2.5_, is the predominant pollutant and has been consistently reported to exceed national and international air quality guidelines in urban–industrial settings [25,26,27]. Importantly, air pollution levels in this region exhibit marked seasonal variation. PM_2.5_ concentrations typically peak during the dry and cool season (November to February), when meteorological conditions such as low wind speed, temperature inversion, and reduced atmospheric mixing height promote pollutant accumulation. In addition, regional biomass burning contributes to episodic haze events during this period [28,29]. In contrast, air quality generally improves during the rainy season (June to October) due to enhanced atmospheric dispersion and wet deposition of airborne particles [29,30]. These seasonal fluctuations may have influenced exposure levels during the study period and should be considered when interpreting the observed prevalence of respiratory abnormalities.

The prevalence of abnormal lung function observed in our study is comparable to that reported in other occupationally exposed populations, where rates range from approximately 4.3% to 56.6% [24,31,32,33,34,35], depending on exposure intensity and diagnostic definitions. The predominance of airway obstruction and restrictive defects reflects the heterogeneous respiratory effects of industrial exposures, including irritant gases, organic and inorganic dusts, and chemical aerosols.

Importantly, when considering pollutants, dust, and airborne contaminants, their chemical composition plays a critical role in determining respiratory toxicity and disease pathogenesis. Particulate matter (PM), particularly PM_2.5_, is a complex mixture that may contain transition metals (e.g., iron, nickel), polycyclic aromatic hydrocarbons (PAHs), sulfates, nitrates, and organic carbon fractions, all of which can induce oxidative stress and airway inflammation [36,37,38]. Inorganic dusts such as silica and asbestos are well known to promote fibrotic lung disease through persistent activation of alveolar macrophages and fibroblasts [3,39], while organic dusts (e.g., endotoxin-containing bioaerosols) can trigger airway inflammation and bronchial hyperresponsiveness [40]. Additionally, VOCs and chemical sensitizers commonly encountered in industrial environments—such as isocyanates, epoxy resins, and acid anhydrides—have been strongly associated with occupational asthma via immunologic and non-immunologic mechanisms [6,8,41]. Therefore, incorporating information on the chemical composition of airborne exposures is essential for understanding their differential contributions to airway obstruction, restriction, and asthma pathophysiology.

Our findings are consistent with large-scale epidemiological evidence demonstrating the adverse respiratory effects of occupational exposure to vapors, gases, dusts, and fumes (VGDF). A COPD surveillance study in southern China involving more than 7000 participants reported significant associations between VGDF exposure and chronic bronchitis, respiratory symptoms, and impaired lung function, particularly reductions in FEV_1_/FVC and FEF_25–75_, indicative of airflow limitation and small airway dysfunction [42]. These spirometric abnormalities parallel those observed in our study population.

Participants with abnormal pulmonary function in our cohort had higher rates of cough, wheezing, and chest tightness, closely mirroring symptom patterns reported in the Chinese study, where cough and sputum production were strongly linked to dust and combined VGDF exposure [42]. Consistently, 6.2% of our participants exhibited small airway disease, highlighting the vulnerability of peripheral airways to inhalational exposures. Importantly, dual VGDF exposure in the Chinese cohort conferred a greater risk of chronic bronchitis and lung function decline than single exposures, suggesting synergistic effects of mixed pollutants. This aligns with our findings that chemical industry employment and male sex were independently associated with abnormal pulmonary function. Together, these data reinforce the role of occupational and environmental exposures in the development of airflow limitation and support early spirometric screening and preventive strategies in industrial and peri-industrial populations.

Restrictive defects accounted for nearly 10% of participants, a finding consistent with prior studies linking long-term occupational exposure to fibrogenic dusts and chemical fumes with reduced lung volumes, even in the absence of overt interstitial lung disease on chest radiography (3,19). The presence of small airway disease in 6.2% of participants further highlights the potential for early peripheral airway involvement, which may precede clinically apparent COPD or asthma [43,44] and is often under-recognized in routine spirometric screening [45].

The prevalence of COPD (4.3%) in our cohort is consistent with population-based estimates from Thailand, which have reported COPD prevalence ranging from 3.7% to 8.3% among adults [5,10,46]. Notably, this burden was observed despite a relatively modest proportion of current or former smokers (18.2%), suggesting that non-smoking-related exposures—such as occupational and environmental pollutants—may play an important contributory role in this population [47,48,49,50].

Asthma prevalence in our study was 5.4%, which falls within the reported range for the Thai adult population (3–10%) [4,5]. However, the significantly higher prevalence of asthma history among participants with abnormal pulmonary function, together with its strong independent association in multivariable analyses, supports the growing recognition of adult-onset and occupational asthma as a major health concern in industrial settings [6,7,8]. Occupational asthma is estimated to account for 5–25% of adult-onset asthma cases globally, and exposure to chemical sensitizers such as epoxy resins and acid anhydrides—commonly encountered in industrial environments—has been well documented [2,6,8].

In our study, multivariable logistic regression analysis identified older age, male sex, a history of asthma, and chest tightness as independent predictors of abnormal pulmonary function. The association with age is consistent with physiological declines in lung function and cumulative exposure to environmental and occupational hazards over time [9,11]. Male sex remained a significant risk factor even after adjustment, likely reflecting sex-based differences in occupational roles, exposure intensity, and smoking patterns, as reported in previous occupational health studies [1].

A prior history of asthma conferred a more than sevenfold increase in the risk of abnormal lung function. This finding aligns with evidence that chronic airway inflammation and repeated exacerbations can lead to airway remodeling, fixed airflow limitation, and overlap phenotypes of asthma and COPD later in life [6,23]. Chest tightness, although reported by a small proportion of participants, emerged as a strong clinical predictor, highlighting the importance of symptom-based screening in identifying individuals at risk for underlying functional impairment [51,52].

Abnormal findings on chest radiography were observed in 8.6% of participants, most commonly lung fibrosis and pulmonary nodules. While radiographic abnormalities were less frequent than spirometric impairments, this discrepancy emphasizes that functional abnormalities may precede structural changes detectable by conventional imaging [3,21]. The combined use of spirometry and chest radiography therefore provides complementary information for early detection and risk stratification in occupational and community screening programs.

The findings of our study have important public health implications for industrial zones in Thailand and other low- and middle-income settings. The substantial prevalence of abnormal pulmonary function among both workers and residents suggests that respiratory risk extends beyond the workplace and may be influenced by ambient air pollution, traffic emissions, and regional biomass burning, all of which contribute to elevated PM_2.5_ levels in central Thailand [1,9]. Early detection through community-based screening programs, as demonstrated in this study, offers an opportunity for timely intervention, smoking cessation, exposure reduction, and appropriate long-term management to prevent disease progression and exacerbations [11,12,51,52].

Key strengths of our study include the use of standardized spirometry following international guidelines, application of GLI reference equations, and comprehensive assessment of symptoms, occupational exposure, and radiographic findings. However, several limitations should be acknowledged. The cross-sectional design precludes causal inference, and exposure assessment relied primarily on occupational categories rather than quantitative exposure measurements. The cross-sectional design limits assessment of disease progression over time, as respiratory conditions such as COPD and asthma develop gradually with cumulative exposures. Longitudinal studies are needed to better understand temporal changes, establish causal relationships, and characterize the natural history of these diseases. In addition, future studies should consider evaluating the occurrence of other comorbidities over time, such as malignancies and neurodegenerative disorders, which may be associated with long-term environmental and occupational exposures. Furthermore, future research should be complemented by approaches that investigate underlying mechanisms at the molecular level, such as multi-omics analyses using non-invasive samples (e.g., blood or urine) or semi-invasive samples (e.g., bronchoalveolar lavage), to better elucidate biological pathways linking environmental exposures to respiratory disease. Additionally, the relatively low prevalence of BDR may underestimate asthma prevalence, particularly in individuals with well-controlled disease or intermittent symptoms. The definitions of COPD and asthma used in this study warrant cautious interpretation. However, based on standard spirometric criteria, reliance primarily on spirometry and BDR may lead to misclassification. In an industrial setting, limiting exposure assessment to cigarette smoking and biomass fuel use may underestimate the contribution of industrial pollutants to COPD burden. Moreover, BDR alone has limited specificity for asthma, as it may be present in COPD and absent in some asthma phenotypes. Without additional objective measures, such as fractional exhaled nitric oxide (FeNO) or bronchial provocation testing, differentiation between COPD and asthma remains uncertain. Therefore, the observed findings may more appropriately reflect airflow obstruction rather than distinct diagnostic categories. Future studies incorporating comprehensive diagnostic tools and detailed assessment of industrial exposures are needed to improve disease classification and clarify exposure–response relationships. Spirometry, while suitable for epidemiological studies, has inherent limitations. A reduced FVC in the presence of a preserved FEV_1_/FVC ratio may reflect either true restriction or airflow limitation with air trapping, and cannot be reliably distinguished without lung volume measurements. Lastly, the lack of longitudinal follow-up limits assessment of disease evolution. Future longitudinal studies are needed to assess temporal changes in lung function, radiographic findings, and clinical outcomes over time in this population.

## 5. Conclusions

In conclusion, abnormal pulmonary function is common among workers and residents of the Navanakorn Industrial Zone, with COPD and asthma prevalence comparable to national estimates yet occurring in the context of significant occupational and environmental exposures. Older age, male sex, a history of asthma, and respiratory symptoms were key determinants of impaired lung function. These results highlight the need for integrated occupational and community respiratory health surveillance, early screening, and targeted preventive strategies in industrial regions of Thailand.

## Figures and Tables

**Table 1:** Baseline characteristics of participants.

Characteristics	Total (n = 373)	Normal Lung Function (n = 258)	Abnormal Lung Function (n = 115)	*p*-Value
Age, years	55.0 ± 13.6	53.9 ± 13.8	57.5 ± 13.0	0.019
Sex				0.002
Female	244 (65.4)	182 (70.5)	62 (53.9)	
Male	129 (34.6)	76 (29.5)	53 (46.1)	
Body mass index, kg/m^2^	25.0 ± 4.4	24.7 ± 4.1	25.5 ± 5.1	0.123
Current or former smoker	68 (18.2)	38 (14.7)	30 (26.1)	0.003
Smoking exposure, pack-years	14.6 ± 16.8	10.9 ± 18.3	19.0 ± 14.1	0.063
Type of factory work				0.037
Textile industry	33 (8.8)	26 (10.1)	7 (6.1)	
Chemical industry	22 (5.9)	10 (3.9)	12 (10.4)	
Other industries	131 (35.1)	87 (33.7)	44 (38.3)	
Non-factory occupations	187 (50.1)	135 (52.3)	52 (45.2)	
Pre-existing comorbidities				
Hypertension	108 (29.0)	66 (25.6)	42 (36.5)	0.031
Dyslipidemia	79 (21.2)	49 (19.0)	30 (26.1)	0.121
Diabetes mellitus	34 (9.1)	21 (8.1)	13 (11.3)	0.327
Coronary heart disease	11 (2.9)	7 (2.7)	4 (3.5)	0.743
Cerebrovascular disease	6 (1.6)	4 (1.6)	2 (1.7)	1.000
Obesity	5 (1.3)	4 (1.6)	1 (0.9)	1.000
Allergic rhinitis	40 (10.7)	23 (8.9)	17 (14.8)	0.091
Asthma	11 (2.9)	2 (0.8)	9 (7.8)	<0.001
COPD	2 (0.5)	1 (0.4)	1 (0.9)	0.522
Respiratory symptoms				
Presence of respiratory symptom	135 (36.2)	80 (31.0)	55 (47.8)	0.002
Cough	67 (18.0)	36 (14.0)	31 (27.0)	0.003
Dyspnea	25 (6.7)	14 (5.4)	11 (9.6)	0.106
Sputum production	58 (15.5)	34 (13.2)	24 (20.9)	0.051
Wheezing	7 (1.9)	1 (0.4)	6 (5.2)	0.002
Chest tightness	9 (2.4)	2 (0.8)	7 (6.1)	0.003
Nasal obstruction	33 (8.8)	23 (8.9)	10 (8.7)	0.324
Rhinorrhea	29 (7.8)	18 (7.0)	11 (9.6)	0.220
Sore throat	13 (3.5)	7 (2.7)	6 (5.2)	0.152
Previous treatment for dyspnea	24 (6.4)	15 (13.0)	9 (3.5)	<0.001
ED visit for dyspnea in the past year	7 (1.9)	2 (0.8)	5 (4.3)	0.009

Data presented as n (%) or mean ± SD. COPD = chronic obstructive pulmonary disease, ED = emergency department, kg = kilogram, m = meter.

**Table 2:** Pulmonary function data and diagnosis of airway disease.

Variable	Data (n = 373)
Spirometry data	
FVC, L	2.79 ± 0.79
FVC, %predicted	94.8 ± 14.1
FVC change after BD test, %	0.46 ± 4.98
FEV_1_, L	2.24 ± 0.67
FEV_1_, % predicted	92.5 ± 14.1
FEV_1_ change after BD test, %	3.04 ± 4.19
FEV_1_/FVC, %	80.41 ± 6.93
FEV_1_/FVC, % predicted	97.56 ± 7.73
PEF, L/s	6.52 ± 1.79
PEF, % predicted	98.77 ± 18.45
FEF_25–75_, L/s	2.20 ± 1.00
FEF_25–75_, %predicted	87.81 ± 31.59
Diagnosis of airway disease	
COPD	16 (4.3)
Asthma	20 (5.4)

Data presented as n (%). BD = bronchodilator response, COPD = chronic obstructive pulmonary disease, FEV_1_ = forced expiratory volume in 1 s, FVC = forced vital capacity, FEF_25–75_ = forced expiratory flow at 25–75% of FVC, PEF = peak expiratory flow, L = liters, s = second.

**Table 3:** Abnormal pulmonary function and chest radiographic findings.

Characteristics	Data (n = 373)
Abnormal pulmonary function	115 (30.8)
Restrictive defect	36 (9.7)
Airway obstruction	48 (12.9)
Mixed obstructive and restrictive defect	8 (2.1)
Small airway disease	23 (6.2)
Bronchodilator response	9 (2.4)
Abnormal chest radiographic findings	32 (8.6)
Lung fibrosis	13 (3.5)
Pleural fibrosis	8 (2.1)
Lung nodule	18 (4.8)
Mixed pattern	15 (4.0)

Data presented as n (%), Airway obstruction defined as FEV_1_/FVC ratio < LLN, Restrictive defect defined as FEV_1_/FVC ratio > LLN and FVC < LLN, Mixed obstructive and restrictive defect defined as FEV_1_/FVC ratio < LLN and FVC < LLN, Small airway disease defined as FEF_25–75_ < 65%, while normal FEV_1_, FVC and FEV_1_/FVC ratio, BDR defined as increase in FEV_1_ or FVC > 10% of the predicted value after bronchodilator responsiveness test, FEV_1_ = forced expiratory volume in one second, FVC = forced vital capacity, FEF_25–75_ = forced expiratory flow at 25–75% of FVC, LLN = lower limit of normal.

**Table 4:** Logistic regression analysis for factors associated with abnormal pulmonary functions.

Variables	Adjusted Odds Ratio (95%CI)	*p*-Value
Age for every 1-year increase	1.026 (1.008–1.045)	0.005
Male sex	2.139 (1.322–3.459)	0.002
History of asthma	7.155 (1.314–38.959)	0.023
Previous treatment for dyspnea	2.440 (0.909–6.555)	0.077
Wheezing	8.326 (0.844–82.140)	0.070
Chest tightness	6.274 (1.188–33.142)	0.031

## Data Availability

The original contributions presented in this study are included in the article. Further inquiries can be directed to the corresponding author.

## Abbreviations

The following abbreviations are used in this manuscript:

BDR	bronchodilator responsiveness
CI	confidence interval
COPD	chronic obstructive pulmonary disease
FEF_25–75_	forced expiration flow rate at 25–75% of FVC
FEV_1_	forced expiratory volume in one second
FVC	forced vital capacity
GLI	Global Lung Function Initiative
L	liter
LLN	lower limit of normal
L/s	liter per second
OR	odds ratio
PEF	peak expiratory flow
VGDF	vapors, gases, dusts, and fumes
